# An Early Stage Diffuse B-Cell Lymphoma Within a Visible Site of Bronchofiberscope Accompanied by Peripheral Lung Cancer

**DOI:** 10.1155/DTE.6.179

**Published:** 2000

**Authors:** Hideaki Shimatani, Kinnya Furukawa, Yoshirou Ebihara, Hiromi Serizawa, Masahiro Tsuboi, Akihiko Ogata, Chimori Konaka, Harubumi Kato

**Affiliations:** 1 Department of 1st Surgery Tokyo Medical University 6-7-1, Nishishinjyuku Shinjyuku-ku Tokyo 160-0023 Japan; 2 Department of Pathology Tokyo Medical University 6-7-1, Nishishinjyuku Shinjyuku-ku Tokyo 160-0023 Japan

## Abstract

We report a case of non-Hodgkin's lymphoma found at the orifice of right B2 accompanied by peripheral lung cancer in a 66-year-old asymptomatic man. Chest X-ray films showed a mass shadow in the left lower lung field. Transbronchial lung biopsy of left S9 demonstrated squamous cell carcinoma. Simultaneously wall thickening at the orifice of the right B2 was found coincidentally. The biopsy specimen from that site showed non-Hodgkin's lymphoma (diffuse B-cell type). After left lower lobectomy, systemic chemotherapy was performed. It is rare for malignant lymphoma to be recognized bronchofiberscopically.


*Diagnostic and Therapeutic Endoscopy*, Vol. 6, pp. 179–182
Reprints available directly from the publisher
Photocopying permitted by license only

© 2000 OPA (Overseas Publishers Association) N.V.
Published by license under
the Harwood Academic Publishers imprint,
part of The Gordon and Breach Publishing Group.
Printed in Malaysia.

### An Early Stage Diffuse B-Cell Lymphoma within a Visible Site of Bronchofiberscope Accompanied by Peripheral Lung Cancer

HIDEAKI SHIMATANI[a,*], KINNYA FURUKAWA[a], YOSHIROU EBIHARA[b], HIROMI SERIZAWA[b], MASAHIRO TSUBOI[a], AKIHIKO OGATA[a], CHIMORI KONAKA[a] and HARUBUMI KATO[a]

[a]*Department of 1st Surgery,* [b]*Department of Pathology, Tokyo Medical University, 6-7-1, Nishishinjyuku, Shinjyuku-ku, Tokyo 160-0023, Japan*

*(Received 12 January 2000; In final form 7 March 2000)*

**We report a case of non-Hodgkin's lymphoma found at the orifice of right B2 accompanied by peripheral lung cancer in a 66-year-old asymptomatic man. Chest X-ray films showed a mass shadow in the left lower lung field. Transbronchial lung biopsy of left S9 demonstrated squamous cell carcinoma. Simultaneously wall thickening at the orifice of the right B2 was found coincidentally. The biopsy specimen from that site showed non-Hodgkin's lymphoma (diffuse B-cell type). After left lower lobectomy, systemic chemotherapy was performed. It is rare for malignant lymphoma to be recognized bronchofiberscopically.**

*Keywords:* BALT, Lung, Non-Hodgkin's lymphoma, Triple malignant disease

### 1. INTRODUCTION

Endobronchial involvement of malignant lymphoma is extremely rare [1], and primary lymphomas in pulmonary parenchymal tissue account only for 0.45% of all pulmonary malignancies [2]. Thus, we report a case of malignant lymphoma that was entirely visible bronchoscopically and was accompanied by peripheral lung cancer.

### 2. CASE REPORT

A 66-year-old man who suffered from laryngeal cancer underwent microsurgery in October, 1997 followed by radiotherapy. The histologic type was moderately differentiated squamous cell carcinoma and the clinical stage was T1aN0M0. In August, 1999, he was referred to our department for detailed examinations for an abnormal shadow on chest

* Corresponding author. Tel.: +81-3-3342-6111. Fax: +81-3-3349-0326.

X-ray film (Fig. 1). His past history was noncontributory apart from his laryngeal cancer. He had never smoked and was asymptomatic. Physical examinations, including lymphonode palpation, showed no abnormalities. A complete blood count revealed; hemoglobin, 14.0 g/dl; leukocyte count, $9.2 \times 10^3/mm^3$ with 70.6% neutrophils, 1.8% eosinophils, 0.2% basophils, 21.3 lymphocytes and 6.1% monocytes; platelet count $206 \times 10^3/mm^3$. Serum

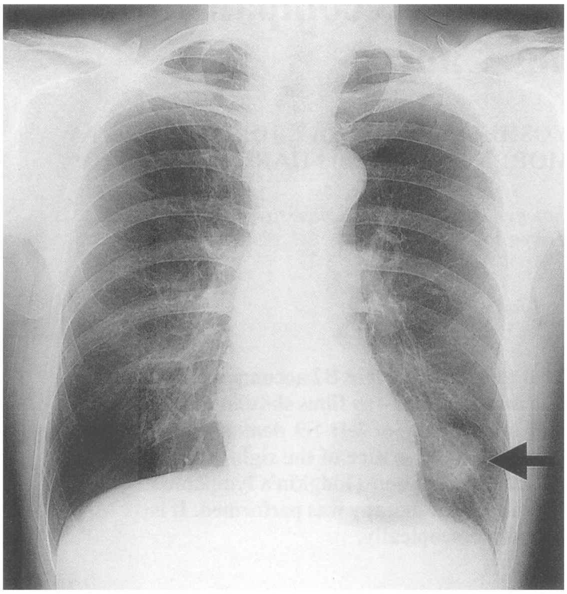

FIGURE 1 Chest X-ray film on admission showing a mass shadow in the left lower lung field (arrow).

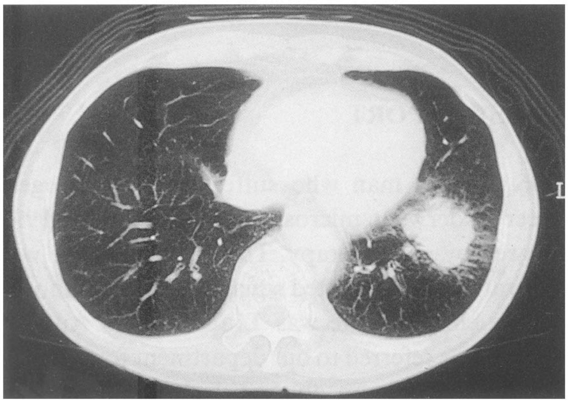

FIGURE 2 Chest CT scan showing a tumorous lesion in left S9.

chemistry showed no abnormality, including tumor markers. CT scan revealed an irregular mass in left S9 (Fig. 2). Lymphadenopathy was seen in the hilar and subcarinal regions. To obtain a definitive diagnosis, transbronchial lung biopsy was performed and the biopsy specimen showed a squamous cell carcinoma of the peripheral lung. Simultaneously a 2 × 3 mm area of thickened bronchial wall was found at the orifice of right B2 (Fig. 3). Immunohistochemical staining as well as hematoxylin and eosin staining for the biopsy specimen from this small lesion were performed (Fig. 4). Findings were negative for LCA, cytokeratin, CG-A, anti-CD43 and CD45RO. Since findings were positive for anti-LN-3, anti-CD20 and anti-CD79, a definitive diagnosis of diffuse B-cell type non-Hodgkin's lymphoma was made. A systemic survey, including abdominal and brain CT, bone and Ga-scintigraphy, showed no metastatic lesions. Systemic chemotherapy, using cisplatin and etoposide was performed and was effective not only for carcinoma but also for lymphoma (Fig. 5(a)). After that, a left lower lobectomy with lymph node dissection via a left thoracotomy was performed. No pleural effusion, adhesion or dissemination were seen. Gross pathologic examination of the resected tumor indicated that it measured 2.5 × 2.4 × 2.5 cm

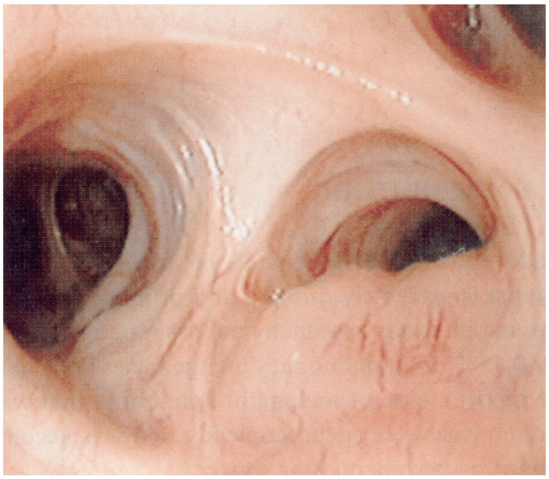

FIGURE 3 Bronchofiberscopy found a wall thickening of the orifice of right B2 unexpectedly.

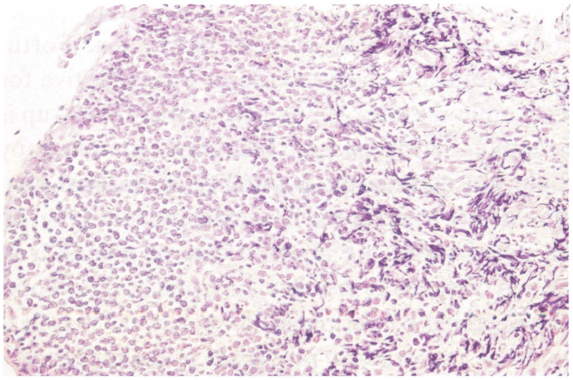

FIGURE 4 Histological stain of the endobronchial lesion shows proliferation of slightly atypical lymphocytes.

in size. The pathological report described a moderately differentiated primary squamous cell carcinoma of left S9. Lymph node metastases were proven in subcarinal, hilar and interlobular nodes pathologically. Adjuvant chemotherapy, consisting of cisplatin, vinorelbine and prednisolone, was added. Bronchoscopy proved complete remission of the lymphoma cytologically and pathologically (Fig. 5(b)).

### 3. DISCUSSION

Most cases of primary pulmonary lymphoma are non-Hodgkin's type. They have a solitary nodule, mass or infiltration on chest X-ray or scanning and seldom involve [3] endobronchial tissue, particularly the region visualized by bronchofiberscopy. In this case, bronchoscopy for the examination of the peripheral cancer unexpectedly discovered a tiny lesion at the orifice of B2 that was negative on the chest radiograph. Immunohistochemical stainings proved that this tumor consisted of B-cell features. A hematoxylin and eosin staining showed proliferation of somewhat atypical and large lymphocytes with notched nuclei. These findings are common to diffuse B-cell type non-Hodgkin's lymphomas. Lymphomas that appear to arise from closely related bronchus-associated lymphoid tissue are known as BALT. BALTs have a tendency to remain localized in the lung for long periods [4]. This case might have been a BALT but we could not prove it, as the biopsy specimen was insufficient to assess centrocyte-like cells or lymphoepithelial lesions [5].

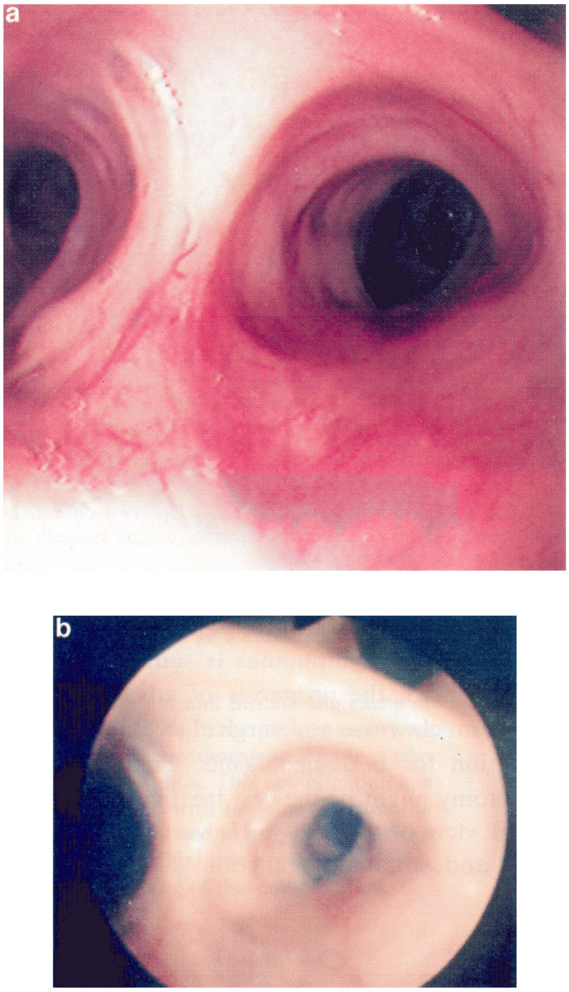

FIGURE 5 Bronchoscopical changes of the lymphoma along with treatments. (a) Pre-operative chemotherapy for the carcinoma was also effective for the lymphoma: a reddening of the mucosa could be observed but the tumor mass had disappeared. (b) No malignancy was proven cytologically or pathologically after two courses of chemotherapy, with redness in the mucosa.

The clinical stage of this case was IE because the diagnostic images showed no abnormality outside the thoracic cavity. Although there is no definition for early stage lymphoma in a strict sense, this case might be considered to be early stage lymphoma because of its bronchoscopical findings.

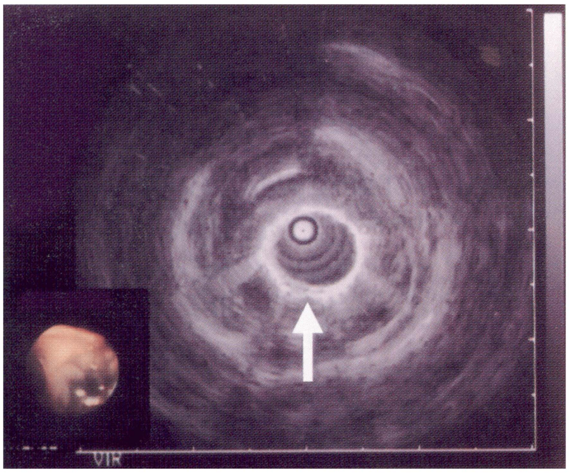

FIGURE 6 Endobronchial echogram after two courses of chemotherapies showing a hypoechoic layer just beneath the mucosa (arrow).

The prognosis of curatively resected primary pulmonary B-cell lymphomas is satisfactory. On the other hand, the prognosis of advanced lung cancer is much worse and surgical treatment is the first option for both neoplasms. Thus, bilateral thoracotomy might be the best treatment from the point of view of curability. However, because of age, PS and pulmonary function, a bilateral procedure was contraindicated. We gave therapeutic priority to the squamous cell carcinoma. Fortunately adjuvant chemotherapies were effective for the lymphoma also. Nevertheless strict follow-up is essential. Endobronchial echogram after therapy could not exclude the possibility of residual lymphoma just beneath the bronchial mucosa (Fig. 6). As stated above, we report a case of minute lymphoma completely visible via a bronchofiberscope, accompanied by peripheral lung cancer. This case might be an extremely rare triple primary disease.

### *References*

[1] Vieta, J.O. and Craver, L.F. Intrathoracic manifestations of the lymphotoid disease. *Radiology* 1941; **37**: 138–159.

[2] Papaioannou, N.A. and Watson, W.L. Primary lymphoma of the lung. An appraisal of its natural history and a comparison with other localized lymphomas. *J. Thorac. Cardiovasc. Surg.* 1965; **49**: 373–387.

[3] Cordier, J., Chailleux, E., Lauque, D. *et al.* Primary pulmonary lymphomas. A clinical study of 70 cases in non-immunocompromised patients. *Chest* 1993; **103**: 201–208.

[4] Herbert, A., Wright, D.H., Isaacson, P.G. *et al.* Primary pulmonary lymphoma of the lung: histopathologic and immunologic evaluation of nine cases. *Him. Pathol.* 1984; **15**: 415–422.

[5] Isaacson, P. and Wright, D.H. Extranodal malignant lymphoma arising from mucosa-associated lymphoid tissue. *Cancer* 1984; **53**: 2515–2524.

